# Managing adolescent eating disorders in primary care: a qualitative study of provider perspectives

**DOI:** 10.1186/s40337-025-01412-w

**Published:** 2025-10-15

**Authors:** Catherine R. Drury, Amanda E. Downey, Siena Vendlinski, Peyton Crest, Pooja Mittal, Erin C. Accurso

**Affiliations:** 1 Department of Psychiatry and Behavioral Sciences, University of California, San Francisco, 675 18th Street, San Francisco, CA 94143 USA; 2 Department of Pediatrics, University of California, San Francisco, 550 16th Street, San Francisco, CA 94148 USA; 3 Department of Psychology, University of South Florida, Tampa, FL USA; 4 Rhodes College, Memphis, TN USA; 5 Health Net, Woodland Hills, CA USA; 6 Philip R. Lee Institute for Health Policy Studies, University of California, San Francisco, San Francisco, CA USA

**Keywords:** Eating disorder, Primary care, Adolescents, Assessment, Treatment

## Abstract

**Background:**

Primary care providers (PCPs) serve a critical role in the identification and treatment of adolescent eating disorders (EDs); yet, few PCPs receive ED training. Without this training, PCPs are not well-equipped to deliver optimal care. Partnering with PCPs is essential to understanding and ameliorating the challenges they face in managing EDs within the primary care setting. This study seeks to explore PCP practices and priorities in the detection and treatment of adolescent EDs to ultimately inform a brief curriculum about EDs for PCPs.

**Methods:**

PCPs in California (*N* = 10) participated in individual, semi-structured interviews focused on a needs assessment, including top priorities in their clinical care setting, current practices around screening and treating EDs, and areas of need around EDs. A multi-step, qualitative thematic analysis process was used to identify and name primary latent themes and subthemes that included both a priori interview content areas and themes uncovered in the data review.

**Results:**

PCPs described structural barriers and resource constraints to supporting ED patients in primary care. Regarding areas for further training, PCPs requested guidance on when to refer to a higher level of care, how to prevent EDs in vulnerable youth, and how to address psychological ED symptoms (e.g., negative body image). Providers reported directly encouraging adolescents with EDs to increase their nutrition, as opposed to utilizing a parent-led approach.

**Conclusions:**

This study adds to the limited literature on the experiences of PCPs in identifying and managing EDs in adolescents. Future research can develop and evaluate strategies for integrating ED screening measures and interventions into PCP workflows, as well as targeted, asynchronous ED training programs for PCPs.

**Supplementary Information:**

The online version contains supplementary material available at 10.1186/s40337-025-01412-w.

## Abstract (plain language summary)

Primary care physicians (PCPs) often serve as a first point of contact in identifying eating disorders in adolescents, and frequently remain involved in an adolescent’s eating disorder treatment. Despite their central role, PCPs typically receive very little eating disorder training. This study interviewed PCPs in California about their perspectives and experiences providing care to adolescents with eating disorders, to ultimately inform a larger goal of developing training materials about eating disorders for PCPs. In their interview responses, PCPs described several challenges to caring for adolescents with eating disorders, including limited mental health referral resources and time constraints. They requested training on preventing eating disorders in youth, managing eating disorders on an ongoing basis, and addressing psychological eating disorder symptoms, such as negative body image. They also indicated that a self-paced eating disorder training would be most accessible for them.

**Supplementary Information:**

The online version contains supplementary material available at 10.1186/s40337-025-01412-w.

## Background

Eating disorders (EDs) affect 9% of the United States (US) population [1] and cause serious medical and psychological symptoms that impair functioning and quality of life [2–4]. Between 1990 and 2019, the incidence of adolescent EDs rose by 22.9%, contributing to the global burden of child and adolescent mental health disorders [5]. Early ED treatment is associated with higher and more sustained recovery rates [6, 7]; yet, many adolescents face delays in diagnosis and barriers to accessing specialized ED care (e.g., stigma, financial and geographic barriers) [8–10], increasing the risk for longstanding impairment into adulthood.

Given the medical complications associated with EDs [4], primary care providers (PCPs) often serve an important role in detection and may even serve as the first point of contact [11–13]. In the US, a widespread shortage of specialist mental health providers [14] has led to increased reliance on PCPs for managing mental health concerns [15]. Moreover, because adolescents with EDs frequently struggle to articulate psychological symptoms due to limited awareness and/or symptom minimization [16, 17], parents may first seek medical explanations for the physical changes they observe. Stigma surrounding EDs and other mental health conditions may further influence parental help-seeking [18, 19]. When an ED is identified, PCPs frequently remain involved in ED care, including coordinating referrals and monitoring vital signs, weight, and related medical concerns (e.g., bone health, menstrual status) [11].

Despite the central role of medical providers in identifying and managing adolescent EDs, PCPs typically receive very little ED training. In the US, Mahr and colleagues [20] surveyed 637 accredited residency programs in internal medicine, family medicine, pediatrics, psychiatry, and child and adolescent psychiatry and found that only 19.3% of programs offered an ED rotation, most commonly as an elective experience. While most programs (85.5%) provided formal didactics on EDs, average ED teaching hours were low, ranging from two to five hours across specialties. This paucity of training has subsequently been found to impact providers’ comfort and competence implementing ED screening and intervention in clinical practice. In a national survey of frontline medical providers across the US, 68% of providers (*N* = 260) denied screening for an ED if it was not the presenting concern, and 59% felt that they lacked the skills necessary to intervene with an ED patient [21]. Anderson and colleagues [22] specifically assessed the ED attitudes and knowledge of physician residents and fellows (*N* = 80) in an urban medical center; only 11% reported feeling comfortable managing patients with EDs. Participants also exhibited several important knowledge gaps, including limited familiarity with ED diagnostic criteria and treatment recommendations [22]. In response to this body of work, efforts have been made to disseminate ED training to PCPs to improve comfort, knowledge, and uptake of recommended screening and management practices [23–25].

These challenges have prompted recent initiatives to integrate ED services into primary care and collaborate with providers to better understand the barriers they face in managing EDs within their practices [24, 26]. Qualitative work with PCPs in countries outside the US (e.g., Australia, Canada, United Kingdom) highlights similar limitations in ED training [27–30], as well as structural and systemic barriers (e.g., limited time during appointments, long waitlists for intensive ED treatment) to delivering optimal ED care [31–33]. To our knowledge, only one study has conducted qualitative, ED-related interviews with pediatric PCPs in the US [26], where differences in the structure and accessibility of healthcare services impacted providers’ experiences. In response to questions regarding their needs, preferences, and barriers to managing EDs in primary care, providers described having insufficient time, training, or skill to deliver appropriate ED services. They also endorsed distress associated with the limited referral options for specialized ED treatment and concerns about the feasibility of expanding ED services within primary care, given the many competing priorities (i.e., in screening, patient education) inherent to primary care appointments [26].

Thus, partnering with PCPs is essential to developing training materials and programs that are acceptable, responsive to providers’ needs, and effective in improving ED detection and treatment within the primary care setting [24, 26]. To this end, the current study sought to further explore PCP practices and priorities related to screening and managing adolescent EDs, to ultimately inform a larger goal of developing a brief curriculum about EDs for PCPs. Prior qualitative work conducted in the US has focused on the need and feasibility of developing a primary care-based ED intervention [26]. This study complements these efforts by seeking to identify specific gaps in provider knowledge and training-related resources that could support practice improvement within primary care. Building on previously developed ED training curricula [23–25], the authors sought to further adapt broad-based education on ED screening, assessment, and management to address issues most relevant to PCPs.

## Methods

Participants (*N* = 10) were PCPs licensed in the state of California who spend at least 40% of their time providing direct care to patients, at least half of whom are under the age of 25. All participants had completed their medical training and licensure. Additional inclusion criteria included the ability to speak English and access the internet. The study team conducted recruitment from January through July 2023 with support from Health Net, a large health insurance provider that covers a primarily publicly-insured population in California. Health Net also provides public insurance plans, which lower-income families are eligible to receive. Health Net distributed a brief blurb via their electronic newsletter and website, and the study team also engaged in direct outreach to primary care practices or providers in California. Eligible participants were approached via email and/or telephone after completing an eligibility screener and enrolled upon consenting to study procedures. Participants completed a 20-minute pre-interview survey via Qualtrics that included questions regarding participant demographics, care setting, burnout, and experience and confidence diagnosing and treating EDs. Subsequently, participants engaged in an individual semi-structured 20-minute interview conducted via Zoom.

Interviews took place between March 2023 and July 2023 and were conducted either by the principal investigator (ECA) or co-investigator (AED), both of whom have expertise in ED treatment (clinical psychologist and pediatrician/child and adolescent psychiatrist, respectively). Participants and interviewers were not known to one another. Interviews focused on a needs assessment and included open-ended questions regarding (1) participants’ top priorities in their clinical care setting; (2) participants’ current practices around screening and treating EDs; (3) aspects of ED care that feel uncomfortable or difficult for participants; and (4) information and resources that would improve participants’ ability to care for young people with EDs (see Supplement for interview guide). Interviewers created brief memos of the themes that arose immediately after conducting each interview, which were used in the coding process to place additional context around each interview. The two interviewers conferred every other week to review recent interviews and their impressions of the interview findings. These meetings were used to determine when thematic saturation had been reached. Interviewers felt that thematic saturation had been reached at eight interviews; they each conducted one additional interview, and upon consultation with one another, recruitment was closed when those last interviews did not appear to reveal any new themes [34].

Participants were compensated with a $50 gift card. The Institutional Review Board at the University of California, San Francisco determined that the project was exempt and did not require further IRB oversight given its focus on program evaluation and quality improvement.

### Analysis

Pre-interview survey responses were reported descriptively as frequency counts or means and standard deviations for items rated on a Likert-type scale. Interviews were recorded, professionally transcribed, and quality checked by one of the study team members. Following Braun and Clark’s [35] steps for thematic analysis, the study team engaged in a multi-step analysis process. After reviewing the entire dataset several times (i.e., reading and re-rereading each interview transcript), the coding team (SV, PC, ECA) developed a codebook including a priori interview content areas (e.g., barriers to care, therapeutic approach, ED screening) and themes uncovered in the data review [36]. The two coders (SV, PC) had previously been trained in qualitative thematic analysis and were supervised by the principal investigator throughout the duration of the project. Coding was conducted using Dedoose version 9.0, 2023. SV and PC independently coded two initial transcripts and then met together with the principal investigator to make final revisions to the codebook. Additional coding disagreements were resolved through discussion among investigators until consensus was reached. Through an iterative process of analytic meetings reviewing and revisiting the codes and their content, the coding team identified and named several primary latent themes and subthemes. All coding discrepancies were discussed and revised until the team came to consensus.

## Results

The average age of the sample (*N* = 10) was 48.80 years (*SD* = 42.50). Participants held degrees in Medicine, Osteopathic Medicine, and Nursing, had been practicing for 16.50 years on average, and were spending 85–100% of their time in patient care (*M* = 96%). Data regarding gender, race, and ethnicity were excluded due to small sample size and risk for being potentially identifiable.

Participant pre-interview survey responses are shown in Table 1. Most participants indicated that caring for patients with EDs was extremely important to their own or their organization’s mission. However, half endorsed no to very little formal ED training, and half reported being somewhat to extremely unconfident in the diagnosis of EDs. Less than half screened for EDs in their practices. Participant burnout level was moderate, with caring for patients with EDs slightly to moderately contributing to burnout for most participants. There was a large spread in the extent to which providers served patients with public insurance (0–85%); half of participants worked in settings where youth with public insurance made up at least 40% of the patient population (*M* = 62%), while the other half worked in settings where 10% or fewer of their patient population were publicly insured youth (*M* = 3%).

**Table 1:** Pre-Interview Survey Results

	*M* (*SD*)	Range
Number of previous ED patients		
Adolescents	28.50 (53.33)	2-175
Young adults	21.30 (46.98)	0-150
Overall burnout level^a^	2.50 (0.85)	
Amount of ED training^b^	3.20 (1.03)	
Confidence diagnosing EDs^a^	2.50 (1.08)	
Confidence treating EDs^a^	2.90 (1.10)	
Importance of caring for patients with EDs		
Importance to organization’s mission^a^	4.30 (0.48)	
Importance to personal or professional mission^a^	4.30 (0.48)	
Extent to which caring for patients with EDs contributes to burnout^a^	2.30 (0.65)	
Feelings about working with patients with EDs^c^	3.80 (0.42)	
Importance of screening for and triaging mental health concerns in practice^a^	4.80 (0.42)	

	*n* (%)	
Management of mental health referrals		
Encourage seeking therapy and treat within practice	1 (10.0%)	
Encourage seeking therapy and place specialty referrals	9 (90.0%)	
Administering any routine mental health screenings	7 (70%)	
Mental health concerns currently screened		
Anxiety	6 (60.0%)	
Depression	7 (70.0%)	
Suicidal ideation/intent	6 (60.0%)	
ADHD	4 (40.0%)	
Substance use	5 (50.0%)	
Adverse childhood events	1 (10.0%)	
Eating disorders	4 (40.0%)	
OCD	1 (10.0%)	

*N* = 10. ED = eating disorder; ADHD = attention deficit hyperactivity disorder; OCD = obsessive compulsive disorder

^a^ Items were rated on a 1 to 5 scale, with higher scores reflecting stronger endorsement of each construct

^b^ Item rated 1 = no formal training or exposure to 5 = extensive training

^c^ Item rated 1 = *dread* to 5 = *enjoy*

### Theme 1: Structural Barriers to Supporting ED Patients in Primary Care

Overall, providers noted many structural barriers and resource constraints to supporting ED patients in primary care. Several providers reported difficulty connecting both ED and non-ED patients to mental health services, which was a repeated source of frustration that contributed to burnout.

*Therapy and counseling*,* as you may be aware*,* has become very*,* very difficult. There are not too many therapists around*,* and if there are*,* they don’t take their insurance. Or if they do take insurance*,* the appointments are like two months out. So*,* therapy has become a very big challenge for us.*

Providers also described having varying amounts of administrative or case management support for facilitating mental health referrals for ED patients. Some reported often taking time during their lunch breaks to call mental health providers for families. They wished for the financial resources to hire ED therapists within their practices or acquire training in mental health interventions for EDs to better meet this need.

*I feel like [integrated care] would be the most beneficial thing. Like if we had…extra money just having an in-house therapist where we can say*,* ‘Come back tomorrow*,* and you’re gonna see our therapist that’s here in the office*,*’ or ‘Come back tomorrow when our psychiatrist is here.’ …you can get started with that—at least get the mental health portion going.*

Providers cited time as being the most limited resource, recognizing that adolescents with EDs and their families often require substantial support. Time constraints are particularly challenging for providers when an ED is unexpected and discovered in the context of a routine annual visit.

*And then*,* unfortunately*,* I think the hardest part is time constraints with us. Like sometimes they’ll come in for a 15-min…visit*,* and I’m like*,* ‘Well*,* this is something*,*’ you know. And then obviously we try to follow up with a longer appointment*,* and then seeing them regularly. But I think time is the biggest problem… caring for these kids.*

Providers attempted to see patients with EDs frequently for follow-up; however, this was often difficult, particularly when scheduling accommodations were made and the adolescent failed to attend the appointment. They highlighted how primary care offices are often not equipped to handle this access pressure.

*I will find a spot on my schedule…. Our schedules are busy*,* and these kids need a lot of follow up until we can get them out to other care…And so these kids—I hate to say ‘clog the schedule*,*’ but they do because… if we’re worried about you*,* I will find a time to see you a week from now….*

Regarding their own competency in treating adolescents with EDs, providers further noted limited time to attend live trainings or read educational materials. Even breaks are often occupied by case management tasks or extra appointments. Providers indicated that self-paced learning formats, such as podcasts or videos, would be most accessible for their schedules.

### Theme 2: Nutrition Counseling at the Intersection of EDs and Obesity

Providers also described barriers to patients accessing nutrition services, both for patients with EDs and those with medical complications associated with higher weight. Although providers have dieticians to whom they can refer families, they noted that health insurance often does not cover nutrition services and parents are reluctant to pay for sessions with a dietician.

*So*,* I actually have a couple of dietitians that have been really great and easy to access*,* but the parents just do not want to spend the money for it […]*,* and ​​a lot of the parents don’t understand how important it is.*

As a result of these challenges, providers reported regularly providing nutrition counseling to patients, with varying approaches. Some cited obesity as the major concern in their practices and endorsed challenges shifting from weight management approaches to providing nutrition counseling appropriate for ED care. For example, while most providers target the types of food being eaten when working with patients with medical complications associated with higher weight, they focus nutrition counseling with ED patients on regular eating throughout the day. Providers endorsed not feeling confident in the nutrition counseling they provide, particularly as pertains to recommending portion sizes.

*I just am really big on promoting fiber and hydration and getting your food groups—your fruits and your veggies—and avoiding the processed crappy sugar*,* and that’s true for everybody. I mean*,* with more overweight*,* I definitely try to target the food groups we don’t want and more of the ones we do want*,* and then with the ones that maybe aren’t*,* food intake issues and more just eating throughout the day*,* it might focus on timing versus type of food. So that might be the difference between there. But talking about portion sizes for me is really hard. I’m not super educated in that and what it should be for everybody’s different body*,* so that’s hard for me to do.*

Although most providers spoke about EDs and obesity as distinct issues, a couple recognized their potential intersection, describing patients who started at a higher weight and then developed a restrictive ED in the context of weight loss efforts. These providers emphasized the importance of minimizing ED risk in any weight-related discussions; for example, by not sharing patients’ weights with them or changing the terminology used.

*I don’t usually give them a number*,* actually*,* since the pandemic*,* with all the kids’ stress. We had families say early in the pandemic*,* ‘Thanks for weighing my daughter at her checkup and telling her what her weight is. Like ever since we were there*,* she won’t eat dinner.’ So*,* actually about two years ago*,* we do blind weights on all our teens. And our staff knows that. They’ll be like*,* ‘What do I weigh?’…And I’m like*,* ‘Let’s talk about other stuff first. You’re more than a number.’*

*Yeah*,* so for the kids who are overweight*,* I usually try to talk to them*,* and I show them their growth chart and say*,* ‘You know*,* your weight is a little bit high.’ I don’t like using any word obese or overweight*,* personally. I know that parents in my history don’t like going back to them [providers who use the words obese/overweight]*,* because*,* you know*,* even though it’s still a medical term*,* they don’t appreciate it. So I try to be like you know*,* let’s try. My goal is usually to focus on three things*,* two being dietary things to change and then one is always exercise. And I try to say small things we can do to change—like one of them might be limiting soda and decreasing the amount of tortillas you eat in a day*,* because that has happened before. You know*,* like if you’re eating or ordering four pieces of bread with breakfast*,* let’s go down to two. Simple basic things for kids who are obese. Usually*,* I try to find like really small things for them to focus on instead of getting overwhelmed.*

### Theme 3: Lack of Standardized ED Screening and Assessment

Providers denied using standardized ED screening measures with their patients but reported that using a brief screening tool would be very or extremely realistic. Although they routinely administered a self-report questionnaire for depressive symptoms (i.e., Patient Health Questionnaire-9), ED assessment was more individualized and based on clinical concern. Some providers described relying on clinical intuition or prioritizing rapport-building with patients, believing that this would encourage disclosure of ED symptoms.

*No*,* I don’t screen everyone for eating disorders right off the bat. I need to get to know the person first*,* and once I get to know the person*,* and we are comfortable with each other*,* and we develop a rapport*,* the patients the majority of the times open up to me*,* saying*,* this is the situation*,* whether it is anorexia or overeating whatever the case*,* and then the approach starts. You don’t talk to somebody with oh*,* the first day you meet them and say*,* ‘Hey*,* you’re depressed*,*’ you know*,* it’s not the approach. Oh*,* every patient of mine is treated as an individual based on their own needs and requirements*,* and so I do not follow any big algorithm or template. There are some guidelines*,* but that’s all they are*,* and I treat them as an individual.*

Other providers noted that their intake forms include questions about dietary habits, and that this information, in combination with regular weight monitoring, guides whether they provide more targeted, ED-related questions.

*Right now we don’t have an official eating disorder screen that we give about for anxiety and depression…So if there’s been any mention or concern in the chart*,* or if I notice big change trends in their growth or their weight on the curve*,* or if they bring it up*,* or if the parent addresses it […]*,* I always do a diet review. And if there’s anything concerning*,* then I’ll delve into it a little bit more. But if their growth is looking good*,* and it seems like they have a reasonable diet*,* and there’s no concern from the parent’s perspective*,* I don’t screen more than that*,* unless I’m worried. Like*,* you know*,* like they’re not having a regular period*,* or a big change*,* or something like that.*

When providers do conduct a more thorough ED screening, they primarily focus on patients’ eating, without assessing for body image concerns specifically. For example, if a patient has lost weight, providers ask specific questions about their diet, in addition to ruling out possible medical causes for weight loss, such as gastrointestinal issues.

*For every physical that we do*,* we have a diet history that we take. We monitor that growth on every visit*,* in fact*,* the eating too. And if we notice the weight is either too low or too high*,* we always follow it on every visit*,* not just the physicals*,* and counsel as much as we can. I mean*,* we don’t use any formal questionnaires. But I just created a little thing on the progress note asking*,* ‘What do you eat? How much do you eat? How often do you eat?’ and go from there.*

Providers who acknowledged negative body image as an important component of EDs expressed discomfort talking about body image with their patients. They requested guidance or strategies to support body image-focused conversations with adolescent patients that emphasize ED prevention.

*One thing I would just say is like in the preteen*,* or how to talk to teenage girls… Like preemptive things to do*,* I guess*,* if you notice any bad input that’s there from your standpoint. […] Yeah some girls make a face at their weight*,* I definitely see that sometimes*,* or they’ll say*,* ‘Oh*,* I weigh too much’ or ‘I’m fat.’ And I’ll be like*,* ‘No.’ And I show them their growth chart and say*,* ‘See*,* it’s my job to tell you if your weight’s too high’ etcetera etcetera*,* especially in the girls who aren’t [at too high of a weight]. [It would be helpful to know] if there’s anything*,* to nip it in the bud*,* besides me just being like*,* ‘No*,* your weight is healthy.’*

Several providers reported challenges during the screening process when concerns were identified based on weight, but adolescents denied disordered eating, minimized their symptoms, and/or engaged in behaviors to conceal the ED (e.g., drinking large amounts of water prior to weigh-ins).

*So*,* I find that adolescents are just difficult in general*,* and they sometimes talk a very good game*,* right? So getting an eye*,* and my training in mental health is not great enough to go underneath that. I had a parent guardian tell me the other day*,* ‘She talks a really good talk*,* and she makes you think everything’s fine*,* but she’s not fine.’ Like when she talks to me*,* she’s like*,* ‘Oh*,* I don’t really have a problem with this. Oh*,* yes*,* sometimes I eat a little bit less.’ I’m like*,* clearly not the case*,* right?*

### Theme 4: Knowledge Gaps in Providing Ongoing ED Care

Most providers reported feeling comfortable providing initial feedback upon identifying an ED, including evaluating vital signs and serum blood markers to determine the need for medical hospitalization, communicating their concern to patients and families, and providing referrals to specialty ED clinics. However, they expressed discomfort providing ongoing care to ED patients at subsequent visits. Indeed, only one participant reported managing medical complications of EDs themselves; other participants referred patients to another provider/practice specializing in EDs. Particularly for patients awaiting specialized medical and therapy appointments, providers were uncertain about additional interventions beyond monitoring weight, vital signs, and providing nutrition counseling.

*The initial steps I feel pretty good about*,* but then what next? How do I help these patients progress towards actual recovery? Cause I feel like a lot of these patients*,* I’m kind of leaving them with*,* ‘You have an eating disorder. These are the things that we need to do to try to help you get better.’ But then*,* they’re all just very stuck*,* and I don’t know how to move them past that point.*

Providers noted a particular gap in their knowledge regarding when to refer to a higher level of care other than medical hospitalization. They indicated that a decision tree or algorithm would be helpful in clearly delineating when to continue outpatient management versus pursue more intensive treatment.

*The other part [that is difficult] is knowing when to step up care. I don’t feel like for me in particular—as an outpatient pediatrician—it’s hard to know where all the components are. Like*,* when are we okay outpatient? When do we need the next step? What are options for the next step? […] I can say*,* “Okay*,* don’t exercise. We’ll see you in two weeks*,* and we’ll see how your vitals are looking.” I can do that. But when is it time to say*,* ‘You need to go to PHP.’? That part is hard. I just don’t know how to do that smoothly. […] Yeah*,* I can admit - I know when to admit someone to the hospital when they’re struggling. That part’s easy for me—to know that difference. It’s the in-between that’s like*,* where do we go from here?*

When adolescents with EDs are connected to specialized services, providers often want to continue supporting their patients’ treatment but are typically not contacted by their patients’ treatment teams. Participants described relying on their patients or their patients’ parents to provide updates on the treatment plan and expressed wanting more direct communication.

*So*,* how could we work together? And oftentimes*,* they’ll come back in to see me… Probably more so than some others in my office*,* I do a lot of follow up in a few months. I wanna see what the specialist has said to you; I wanna know where we are so that we could work as a team*,* right? And they don’t always come back to me*,* but they often times will… And I would love for*,* in the interim*,* someone to call me and say*,* ‘Hey*,*’ or like*,* send me a message versus like a chart note that I’m not going to necessarily see until I open that patient when they’re in my office*,* right? ‘Hey. This is where we’re at. This is how you can help now.’ So then*,* I can be like*,* okay*,* I can bring this patient in*,* or they’re scheduled with me in my office*,* right? And if the parents really involved*,* then they’ll be the one to do that. But it’s not always the case.*

#### Subtheme 4.1: Experiencing patients with EDs as “frustrating” and “manipulative”

Providers frequently attributed denial, minimization, and concealment of symptoms (common place in the context of EDs) to the young person themselves, without recognizing these responses as intrinsic to an ED.

*I think the part that’s most uncomfortable and this is a little bit of a generalization but a lot of the eating disorder teens that I’ve taken care of can be a little bit controlling and a little bit manipulative…Like they lie about what they’re eating*,* they try to lie out their way like water loading or whatever.*

*They come in*,* and they… act very indifferent. They don’t care*,* or they ask*,* ‘Why do I have to come here all the time?’ …so it’s very frustrating*,* and then we refer them to a specialist.*

Providers reported frustration when adolescents with EDs refuse referrals to mental health therapy or specialty care. Although providers continue to monitor these patients’ weight, they expressed feeling uncertain and overwhelmed as to how to best support them in the absence of specialized treatment.

*Obviously mental health*,* like [I’m] trying to refer them to the appropriate place. A lot of them like refuse*,* and*,* unfortunately from our standpoint*,* there’s not much I can do.*

#### Subtheme 4.2: Llimited use of parents as a resource in ED management

Providers incorporated parent perspectives into ED screening and assessment to varying degrees. Many recognized that parents may be more likely to report ED symptoms that youth do not disclose. At the same time, they also acknowledged that some adolescents may be more forthcoming when parents are not present, creating a tension between fostering rapport with the adolescent and the need to relay certain information (e.g., purging) to parents. Additionally, they highlighted potential benefits of giving parents the opportunity to discuss their child’s eating behavior without the child present.

*I always talk to the parent alone too. Just to get a sense of what’s going on at home*,* what are mealtimes like*,* what are some of your concerns? I always try to have everyone get their time alone and everyone gets their time together because I also don’t think it’s good when the parent is complaining about the kid*,* or like basically outing the kid even further*,* because then sometimes it seems like you’re teaming up with the parent and not them.*

Evidence-based treatments for adolescent EDs (e.g., family-based treatment [FBT]) empower caregivers to support their child in receiving adequate nutrition and proactively managing ED behaviors. In line with this philosophy, providers reported regularly including parents in discussions about concern for an ED, next steps, and recommended referrals. However, direct management of ED concerns (e.g., increasing nutrition, weight gain) were handled very differently, in contrast to FBT principles. Several providers described disordered eating behaviors as a means of adolescents exerting control over their lives. Thus, providers tried to empower adolescents to initiate behavioral change. For example, providers reported trying to reduce dieting behavior by talking with adolescents about healthy eating and disparaging myths about food and eating often seen on social media. Further, providers generally discouraged parents from talking too much about eating or encouraging their child to eat, emphasizing that this kind of parental intervention would likely result in the adolescent exerting greater efforts to restrict their intake.

*Then I’ll go with the appetite stimuli or more of like a high calorie [foods] and talk to them about doing more calorie smoothie shakes*,* you know nothing drastic*,* but something to kind of like think*,* ‘Oh*,* I have control in my food*,*’ because a lot of the eating disorders is like control of their own—they can’t control anything else. They can control the food. In addition to all that*,* I also tell them to make their own food. If they don’t like what mom’s cooking*,* then you make your own.*

*So usually what I do is I first try to emphasize to the parent that food can be a big form of like control*,* and emphasize that they need to be cautious*,* but try not to make a big deal about it*,* or getting a lot of fights because… the adolescent or the early to pre-teen really grabs onto that and uses that as a form of control.*

*…I really just try to emphasize don’t be fighting with your teenager over how much they’re eating or not eating at the beginning.*

## Discussion

This study adds to the limited literature on the experiences and perspectives of PCPs, who serve a critical role in identifying and managing EDs in adolescents. Similar to prior work [26, 31–33], PCPs emphasized several challenges to delivering optimal care to ED patients within the limitations of the healthcare system. Although limited mental health referral resources and time constraints described in the current study and prior literature [26, 31–33] are not necessarily unique to individuals with EDs, the medical complexity of these illnesses, combined with adolescents’ ambivalence about treatment, appear to intensify the impact of structural issues. Particularly when patients face barriers to accessing specialized ED care, providers expressed uncertainty about how to provide ongoing support within the primary care setting, including when to refer to a higher level of care and how to address psychological ED symptoms (e.g., negative body image). This study builds on prior studies in highlighting how structural barriers also hinder PCPs from acquiring greater ED expertise, as they noted insufficient time for trainings and emphasized the need for accessible educational materials (e.g., a decision tree for higher level of care referrals).

ED prevention was also a topic of interest for participants, including guidance on weighing patients, responding to patients’ concerns about their weight, and providing nutrition counseling. Several responses alluded to tension between providers’ training in obesity prevention and management and ED prevention and treatment in their practices, a theme that was not identified in existing PCP needs assessment literature. Prior work examining the role of weight stigma in medical care has found that providers’ well-meaning recommendations for weight loss and dietary changes can precipitate or exacerbate ED symptoms in vulnerable adolescents, highlighting a need for improved training in weight-neutral approaches to medical care [24, 41, 42].

Expanding on prior accounts of PCPs’ limited confidence managing EDs [26, 31], providers described diverse strategies for encouraging adolescents to increase their caloric intake. Some PCPs recommended allowing the adolescent to make their own food choices and accommodating food preferences to reduce short-term distress, but this approach may inadvertently maintain disordered eating behaviors and cognitions and impede necessary weight gain over time. Emerging research has explored the use of FBT principles (e.g., separating the illness from the patient, using metaphors that compare EDs to medical illnesses) within primary care to empower parents to temporarily take charge of their youth’s nutrition and weight restoration [38]. Preliminary evidence supports the feasibility and effectiveness of this model [39, 40], in which PCPs support parents in making recovery-oriented eating decisions for their child and challenging ED-related food preferences and behaviors at home. Thus, integrating FBT-informed education and interventions into training and resources for PCPs may improve their capacity to support ED recovery in the absence of or in conjunction with specialized ED care. Although more research is needed to develop and refine brief ED interventions suitable for the primary care setting, better equipping providers to intervene may help reduce the frustration caused by managing ED cases with minimal improvement.

Overall, any ED training for PCPs should be developed with consideration of the aforementioned structural challenges to support feasibility and uptake (e.g., through on-demand training platforms). ED training curricula also must be strategic in the information provided, given limited time during medical visits and already burdened providers. In response to the current study’s findings, initial ED training materials were adapted to help PCPs maximize their time and expertise by focusing on critical components of primary care (e.g., identifying problematic behaviors or growth patterns) and leaving other tasks (e.g., making a specific ED diagnosis) to other team members. Additionally, study results pertaining to weight management and nutrition in the context of higher weight informed the presentation of a framework for nutrition counseling that emphasizes variety, flexibility, and sufficient density to support a healthy lifestyle. Education was also added regarding preventative approaches to discussing eating and weight with adolescents, such as reinforcing healthy behaviors as opposed to weight loss, and avoiding concepts about foods being ‘healthy’ or ‘unhealthy.’ This was combined with efforts to raise awareness about atypical AN, which can occur in youth at higher weights and is associated with serious medical complications comparable to AN [43]. Adapted training materials further emphasized the role of the family in medical counseling for EDs and best practices for collaborating with mental health providers. Finally, in direct response to provider feedback, an ‘if, then’ flowchart of common ED-related scenarios was developed to support medical management and referrals. These adaptations aim to streamline ED-related care in primary care settings by equipping providers with practical tools that reflect both the demands of modern clinical practice and the nuanced needs of youth with EDs.

The strengths of this study include its use of qualitative methods to interview PCPs with a wide range of years’ experience in clinical practice, whose perspectives on adolescent EDs are underrepresented in the current literature. While findings have the potential to inform an ED training program for PCPs, the experiences of participants may not generalize to all providers working in primary care settings, particularly those in different geographic locations and healthcare systems. Specifically, all study participants provided care within the state of California and were relatively homogenous in terms of gender, race, ethnicity, and care setting. Although participants reported that up to 85% of their patients had public insurance, the average proportion of publicly insured patients was only 32%, such that the majority of patient care experiences were with privately insured patients. Further, participants were likely more interested in ED topics given their interest to participate in this study.

Additional study limitations include the small sample size, particularly given the heterogeneity in provider types (e.g., doctor of medicine, nurse practitioner) within the sample. Physicians and nurses generally receive very different training, which may have obscured themes that would have been more prominent in a sample of only physicians or nurses. Finally, additional details about the settings in which participants practice (e.g., billing structure, access to ED specialty care referral options) were not collected and could provide additional context for providers’ perspectives and practices in caring for ED patients.

## Conclusion

As a first point of contact for many young people with EDs, primary care settings are well-positioned to improve ED detection rates and increase access to appropriate ED services. Partnering with PCPs is essential to understanding the clinical and structural barriers that limit their effectiveness in caring for adolescents with EDs, and to developing training and interventions that ameliorate these barriers. Future research should evaluate strategies for integrating ED screening and interventions into PCP workflows, as well as the effectiveness of targeted, asynchronous training programs in reaching providers, improving their competence, reducing burnout, and improving patient care. In order to facilitate and reinforce new learning among PCPs, didactic training programs will likely need to be supplemented with consultation and opportunities for practical application. Given rising rates of EDs among adolescents [5] and an insufficient number of specialty providers, greater attention to supporting and equipping PCPs is vital to meeting the growing need.

## Supplementary Information

Supplementary material 1.

## Data Availability

The datasets used and/or analyzed during the current study are available from the corresponding author on reasonable request.

## Abbreviations

ED: Eating disorder
FBT: Family-based treatment
PCP: Primary care provider
US: United States
