# Supplementary material for: Managing adolescent eating disorders in primary care: a qualitative study of provider perspectives
Source: J Eat Disord. 2025 Oct 15;13:223. doi: 10.1186/s40337-025-01412-w (PMC12523073; doi:10.1186/s40337-025-01412-w)
Supplement: Supplementary file 1 — Supplementary material 1. [file 40337_2025_1412_MOESM1_ESM.docx]

**Supplement**

**Interview Guide**

1. What are your top three priorities in your practice related to patient care?
   1. *If mental health or eating disorders (EDs) not on there:* Where does mental health fit in with respect to your clinical priorities? What about EDs?
2. What’s your current practice around identifying young people with EDs?
   1. *Only screen if red flag, or more globally?*
   2. What are your next steps if you’re concerned about a potential ED?
      1. *If needed:* What kind of counseling might you provide?
      2. *If needed:* What referrals/labs/next steps would you take?
         1. *What results would you be most worried about?*
         2. *Any indicators that would make you think about medical hospitalization?*
      3. What can get in the way of taking these next steps?
         1. *If needed:* Are there other things you would want to do in these visits to assess out an ED if time wasn’t a constraint?
3. What aspects of caring for young people with EDs are difficult or feel uncomfortable for you?
4. What kind of information would be most valuable to you in caring for young people with EDs?
   1. Any specific kind of information that would help to improve gaps in your knowledge?
   2. Resources?
